# The neural substrates of risky rewards and losses in healthy volunteers and patient groups: a PET imaging study

**DOI:** 10.1017/S0033291720005450

**Published:** 2022-10

**Authors:** Nikolina Skandali, Joonas Majuri, Juho Joutsa, Kwangyeol Baek, Eveliina Arponen, Sarita Forsback, Valtteri Kaasinen, Valerie Voon

**Affiliations:** 1 Department of Psychiatry, University of Cambridge, Cambridge, UK; 2 Cambridgeshire and Peterborough NHS Foundation Trust, Cambridge, UK; 3 NIHR Biomedical Research Centre, University of Cambridge, Cambridge, UK; 4 Department of Neurology and Turku Brain and Mind Center, University of Turku, Turku, Finland; 5 Turku PET Centre, University of Turku, Turku, Finland; 6 Department of Neurology, Päijät-Häme Central Hospital, Lahti, Finland; 7 Division of Clinical Neurosciences, Turku University Hospital, Turku, Finland

**Keywords:** Risk-taking, PET imaging, serotonin, opioids

## Abstract

**Background:**

Risk is an essential trait of most daily decisions. Our behaviour when faced with risks involves evaluation of many factors including the outcome probabilities, the valence (gains or losses) and past experiences. Several psychiatric disorders belonging to distinct diagnostic categories, including pathological gambling and addiction, show pathological risk-taking and implicate abnormal dopaminergic, opioidergic and serotonergic neurotransmission. In this study, we adopted a transdiagnostic approach to delineate the neurochemical substrates of decision making under risk.

**Methods:**

We recruited 39 participants, including 17 healthy controls, 15 patients with pathological gambling and seven binge eating disorder patients, who completed an anticipatory risk-taking task. Separately, participants underwent positron emission tomography (PET) imaging with three ligands, [^18^F]fluorodopa (FDOPA), [^11^C]MADAM and [^11^C]carfentanil to assess presynaptic dopamine synthesis capacity and serotonin transporter and mu-opioid receptor binding respectively.

**Results:**

Risk-taking behaviour when faced with gains positively correlated with dorsal cingulate [^11^C]carfentanil binding and risk-taking to losses positively correlated with [^11^C]MADAM binding in the caudate and putamen across all subjects.

**Conclusions:**

We show distinct neurochemical substrates underlying risk-taking with the dorsal cingulate cortex mu-opioid receptor binding associated with rewards and dorsal striatal serotonin transporter binding associated with losses. Risk-taking and goal-directed control appear to dissociate between dorsal and ventral fronto-striatal systems. Our findings thus highlight the potential role of pharmacological agents or neuromodulation on modifying valence-specific risk-taking biases.

## Introduction

### Decision making under risk

Risk evaluation is a central component of daily decisions, whether it refers to minor, seemingly inconsequential, or major life-changing decisions. Risk is commonly defined as a known probabilistic variation in the distribution of outcomes (Weber, Shafir, & Blais, 2004). Most individuals are typically risk-averse preferring safer options, but this preference is influenced by several factors including inter-individual differences, past personal experiences and delay in the receipt of reward (Cardinal, 2006). Prospect theory suggests that when confronted with decisions entailing risk, humans apply a fourfold pattern of decision-making which varies as a function of the nonlinear weighting of probabilities, and valence of potential outcomes, i.e. gains *v.* losses (Kahneman & Tversky, 1979).

We have previously shown a critical role for outcome valence using a computer task assessing risk tendencies between certain and gamble options and dissociating reward and loss anticipation (Voon et al., 2015). Binge eating disorder (BED) subjects, similar to methamphetamine-dependent and alcohol-dependent subjects, have greater risk-taking to rewards whereas obese subjects without binge eating show higher risk-taking for high-probability small losses (Voon et al., 2015). Binge drinkers make more risky choices when faced with high-risk losses mediated by diminished sensitivity to the anticipation of high-risk negative outcomes (Worbe et al., 2014). We have also shown that deep brain stimulation of the subthalamic nucleus decreases risk-taking to rewards but acute stimulation decreases the capacity to discriminate loss magnitude (Voon et al., 2018).

### Neurochemical substrates of decision-making under risk

Several brain areas including the anterior cingulate cortex, ventromedial prefrontal cortex, the insular and parietal cortex, and ventral striatum are implicated in distinct aspects of risk-taking behaviour including outcome anticipation, reward coding and uncertainty of outcomes (Christopoulos, Tobler, Bossaerts, Dolan, & Schultz, 2009; Critchley, Mathias, & Dolan, 2001; Tobler, O'Doherty, Dolan, & Schultz, 2007). A major neurotransmitter system linked with decision-making under risk is dopamine (DA) (Fiorillo, Tobler, & Schultz, 2003) with the abnormal dopaminergic transmission in the prefrontal-cortical striatal circuitry underlying maladaptive decisions when faced with risks (Simon et al., 2011). In healthy volunteers, the DA precursor L-dopa increases risk-taking behaviour in gambles involving potential gains, but not in loss-only gambles (Rutledge, Skandali, Dayan, & Dolan, 2015). Parkinson's disease (PD) patients make more risky choices in the Iowa gambling task (Kobayakawa, Koyama, Mimura, & Kawamura, 2008), a well-validated task assessing risk preferences by simulating real-life decision making under uncertainty (Bechara, Damasio, Damasio, & Anderson, 1994). Additionally, PD patients with impulse control

disorder make more risky choices and have reduced ventral striatal activity when tested on *v.* off DA agonists (Voon et al., 2011).

While DA is implicated in reward learning and motivation during decision making, endogenous opioids are implicated in the hedonic responses to rewards (Berridge, 1996). In healthy volunteers, naloxone (a competitive, non-selective opioid receptor antagonist) decreases pleasure ratings for larger rewards, reflected by reduced brain activity in the rostral anterior cingulate cortex, and increases aversive ratings for losses of various magnitudes (Petrovic et al., 2008). In rodents, blockade of opioid transmission with naloxone reduces sensitivity to changes in reward value (Wassum, Ostlund, Maidment, & Balleine, 2009) and increases aversive responses, such as conditioned place aversion (Narayanan et al., 2004).

### The role of serotonin in decision-making under risk

Unlike DA, the role of serotonin (5-HT) in risky decision making is less well established. Pathological gamblers show hypoactive 5-HT system (Moreno, Saiz-Ruiz, & López-Ibor, 1991). Animal and human studies show somewhat inconsistent findings of altered 5-HT on risk-taking tendencies. In both primates (Long, Kuhn, & Platt, 2009) and rodents (Koot et al., 2012), decreasing central 5-HT through tryptophan depletion increases risk-taking. In humans, tryptophan depletion reduces the ability to discriminate between reward magnitudes but not losses and does not influence risk tendencies (Rogers et al., 2003). Rats homozygous or heterozygous for serotonin transporter (SERT) knockouts, resulting in higher extracellular 5-HT, perform better in a rodent version of the Iowa gambling task (Homberg, van den Bos, den Heijer, Suer, & Cuppen, 2008). Similarly, the short allele of the serotonin transporter-linked polymorphic region (HTTLPR), linked with reduced SERT expression and function, is associated with suboptimal decision-making in the Iowa gambling task (Homberg et al., 2008), which does not appear to be mediated by changes in sensitivity to probabilistic reinforcement (Mobini, Chiang, Ho, Bradshaw, & Szabadi, 2000). Tryptophan supplement for 14 days in healthy volunteers altered the combined weighting of gains and small losses reflecting a reduction in loss-aversion (Zeeb, Robbins, & Winstanley, 2009). Some of the effects of 5-HT on risk-taking behaviour might be mediated through its role in aversive emotional processing (Cools, Roberts, & Robbins, 2008; Zeeb et al., 2009). Indeed, SERT binding in ecstasy users has been negatively correlated with amygdala activation in response to angry facial stimuli (Laursen et al., 2016).

### Study aims

In this study, we examined the neurochemical correlates of risk-taking as a function of valence across healthy controls and patient groups using three PET ligands to evaluate presynaptic DA function, and mu-opioid receptor and SERT binding. We selected gambling disorder (GD) patients, as risky decision making is central to the GD pathophysiology with increased risk-taking on decision-making tasks including the Iowa gambling task and the Cambridge gamble task (reviewed in Limbrick-Oldfield et al., 2020), and BED patients as this patient group is characterised by increased risk-taking in anticipation of rewards (Voon et al., 2015). We employed a transdiagnostic approach rather than focus on group comparisons, similar to our previous study elucidating the role of these three neural systems in the arbitration between goal-directed and habitual strategies when faced with both gains and losses (Voon et al., 2020). We applied the same risk-taking task we used in previous studies including BED patients and alcohol and methamphetamine-dependent subjects (Voon et al., 2015), obsessive-compulsive disorder patients (Voon et al., 2018) and binge drinkers (Worbe et al., 2014).

## Materials and methods

### Recruitment

We recruited 39 subjects consisting of 17 healthy volunteers (HV), 15 patients with GD and seven BED patients. The study protocol was approved by the Ethics Committee of Hospital District of Southwest Finland. All subjects signed a written informed consent form and the study followed the principles of the Declaration of Helsinki. Inclusion criteria included fulfilling the DSM-IV diagnostic criteria of BED or GD for the corresponding groups. None of the included subjects used serotonergic medications or medication known to have effect on DA or opioid system. As described in detail in previous reports of data from the same cohort (Majuri et al., 2017*a*, *b*), participants were instructed to refrain from smoking cigarettes 8 h prior to scanning, from drinking coffee or tea 12 h prior to scanning, and from drinking alcohol 48 h prior to scanning. They were allowed to eat a normal breakfast prior to the PET scans and a standard hospital lunch was served between scans.

### PET scanning

Participants were scanned using three PET ligands, [^18^F]FDOPA, [^11^C]MADAM and [^11^C]carfentanil for assessing presynaptic DA transmission (Nanni, Fanti, & Rubello, 2007), SERT (Halldin et al., 2005) and mu-opioid receptor (Hirvonen et al., 2009) binding, respectively. To minimize the possible effects of arousal on tracer binding (Li & van den Pol, 2008), subjects were not allowed to sleep in the scanner during [11C]carfentanil imaging.

### PET radioligand synthesis

Radioligands were produced according to EU GMP regulations at the Turku PET Centre, as previously described (Forsback, Eskola, Bergman, Haaparanta, & Solin, 2009; Hirvonen et al., 2009). [^18^F]FDOPA was synthesized via electrophilic radiofluorination. [^11^C]Carfentanil was synthesized via ^11^C-methylation of desmethyl carfentanil (sodium salt) with [^11^C]methyl triflate prepared from cyclotron-produced [^11^C]methane. [^11^C]MADAM was synthesized via the ^11^C-methylation of desmethyl MADAM with [^11^C]methyl triflate prepared from cyclotron-produced [^11^C]methane using a previously described method (Halldin et al., 2005), with minor modifications. Radiochemical purity exceeded 95% in all production runs and the average specific activity was 395 GBq/μmol (s.d. 130) for [^11^C]MADAM, and more than 5 GBq/μmol for [^18^F] FDOPA and 590 GBq/μmol (s.d. 290) for [^11^C]carfentanil at the time of injection.

### PET methodology

PET scans were performed using Siemens High-Resolution Research Tomograph PET scanner (HRRT, Siemens Medical Solutions, Knoxville, TN, USA) in 3D mode with scatter correction. A transmission scan was performed before dynamic scans for attenuation correction and was carried out with a ^137^Cs rotating point source. The dynamic scan was divided into 19 frames (3 × 1 min, 4 × 3 min, 10 × 6 min and 2 × 7.5 min).

The dynamic scanning times were 90 min for [^11^C]MADAM, 51 min for [^11^C]carfentanil and 90 min for [^18^F]FDOPA. All three PET scans were conducted in the same day at fixed intervals: [^11^C]carfentanil scan at 0900–1000 h, regular hospital lunch at 1100–1200 h, [^11^C] MADAM scan at 1200–1300 h, and [^18^F]FDOPA scan at 1430–1530 h. One [^11^C]carfentanil scan and three [^18^F]FDOPA scans were performed on a separate day due to tracer production failure or scanner malfunction. The average injected doses were 495 MBq for [^11^C]MADAM, 494 MBq for [^11^C]carfentanil and 227 MBq for [^18^F]FDOPA. Further details can be found in previous publications from the same cohort (Majuri et al., 2017*a*, *b*).

Head movements were minimised applying a personalised thermoplastic mask or a Velcro strap and recorded using a stereotaxic infrared camera (Polaris Vicra, Northern Digital, Waterloo, Canada) during scanning. Three GD patients and one BED patient had a Velcro strap instead of a thermoplastic mask during [^18^F]FDOPA scanning. Further details can be found in Supplementary materials and methods.

### Data pre-processing

**Fig. 1.**
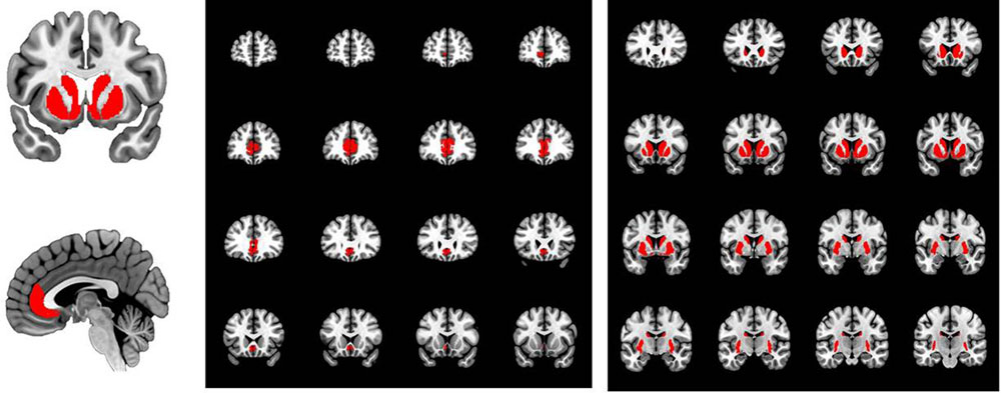
Regions of interest (ROIs) in the striatum and rostral cingulate gyrus as delineated with FreeSurfer software from individual T1-weighted MR images.

The detailed description of the PET data processing is provided in our previous publications (Majuri et al., 2017*a*, *b*). PET images were corrected for between-frame motion and coregistered with individual anatomical T1-weighted magnetic resonance imaging (MRI) using Statistical Parametric Mapping software (SPM8, http://www.fil.ion.ucl.ac.uk/spm/software/spm8/), run on Matlab R2012a (MathWorks, Natick, MA, USA). Regions of interest (ROIs) were delineated with FreeSurfer software (version 5.3.0, http://surfer.nmr.mgh.harvard.edu/) from individual T1-weighted MR images (Figure 1). A simplified reference tissue model was applied to calculate [^11^C]carfentanil and [^11^C]MADAM ratios of specific binding relative to the non-displaceable binding for the selected ROIs (Gunn, Lammertsma, Hume, & Cunningham, 1997). Similarly, a Patlak plot was used to extract [^18^F]FDOPA influx constant rates (Patlak and Blasberg, 1985).

### Task description

**Fig. 2.**
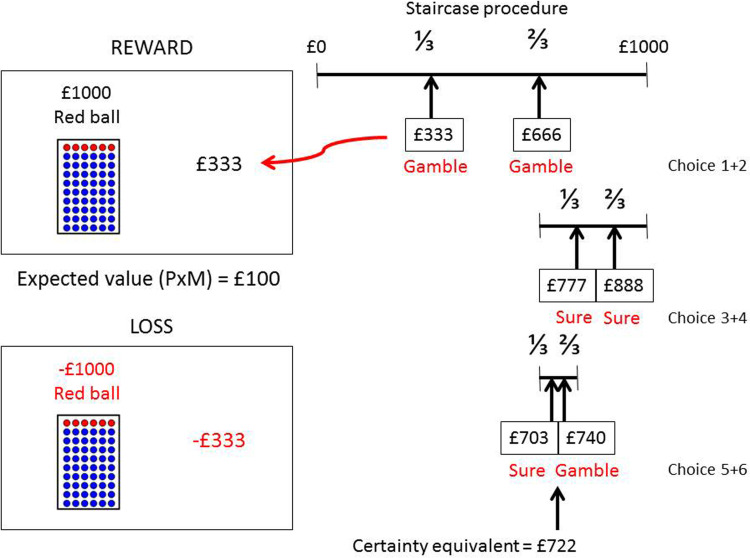
Adaptive risk-taking task. Subjects choose between a risky and sure prospect in which the amount of the sure choice was adapted as a function of whether the subjects chose the sure or gamble amount in order to calculate the certainty equivalent. The task is described in detail in the text.

Participants were separately tested on an anticipatory risk-taking task for reward and loss outcomes. The task (Figure 2), detailed in Voon et al. (2015), involved choosing between a certain option and a gamble in two valence versions; reward and loss. Participants were instructed that if they chose the gamble, the computer would randomly select a ball from the jar filled with red and blue balls. If the ball was red, participants would win (or lose) the specified amount on top of the jar. If the ball was blue, participants would not win (or lose) any amount. The reward probabilities varied on four different levels, *p* = 0.1, 0.3, 0.5, 0.9, represented by the number of red balls (compared to blue ones) in the jar, with five expected values for each level, *E* = £10, £50, £100, £500, £1000, thus giving a total of 20 prospects. The order of the probability level and the expected value was randomised. If participants selected a certain option, they would win (or lose) the amount indicated on the right side of the screen.

Participants were tested separately on the reward and loss versions and the order was counterbalanced across subjects. For each prospect, the certainty equivalent (CE, the certain amount of money that would be accepted instead of a gamble) was computed in a stepwise manner depending on previous choices. The value (*V*) of the gamble (amount indicated over the jar) was calculated as *V* = EV/*P* (i.e. for *p* = 0.1, EV = £100, *V* = £1000). The CE range for each prospect was determined by defining the range of values between 0 and the value of the gamble (e.g., 0 to £1000). In trials 1 and 2, the amount of the certain choice was the one-third and two-third cut-point values. The interval for the next two trials included only the interval accepted by the subject in the first two trials. For example, if the subject rejected the lower and middle third, the upper third was used as the range for trials 3 and 4. The amount of the certain choice was then the one-third and two-third cut-point values of this upper third range. The same process was repeated for trials 5 and 6 and the average of these choices was used to determine the CE.

Participants performed a practice trial of the task with six choices indicating the stepwise method and changes in the magnitude of the certain option, for both reward and loss task versions. The task was self-paced. No feedback was given after the end of each trial. Participants were instructed that at the end of the task, they will gain or lose a proportion of the total amount earned or lost, randomly selected by the computer. The task was coded in e-PRIME, 2.0.

The weighted probability, *w*(p), was calculated according to prospect theory as:
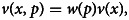
where *v*(*x*, *p*) is the subjective value of amount × at probability *p* (i.e. the CE) and *w* is the decision weight of the objective probability *p*.

The main outcome measure was the average *w*(*p*) for reward and loss which reflects risk-taking propensity.

### Data analysis

Statistical analyses were performed using SPSS (IBM SPSS Statistics, version 22, Armonk, NY, USA). We checked for normality of distribution using Shapiro–Wilk test (*p* > 0.05). Group differences in demographic data and questionnaire data were investigated using an ANOVA model (three groups) or χ^2^ tests for categorical variables. As the [^18^F]FDOPA was not normally distributed, within-group correlation analyses between tracer binding and average reward and loss weightings were performed with the Spearman rank-order test (significance was assigned following multiple corrections using Bonferroni *p* < 0.002).

Since several regions were observed to be correlated with risk-reward weightings, we then asked which regions for the [^11^C]carfentanil were independently associated with reward weightings using partial correlation analysis. The assumptions to enter the data into the analysis were fulfilled (continuous linearly related normally distributed variables without outliers). As risk-taking to loss was associated with striatal [^11^C]MADAM binding, we further sought to ask which components of striatum might be specifically implicated. Similar to the above analyses we first conducted a Spearman rank correlation assessing the relationship between loss probability weighting and ventral striatal, caudate and putamen [^11^C]MADAM binding correcting for multiple comparisons (Bonferroni corrected adjusted *p* value <0.016 was considered significant). We then ran a multiple regression analysis with loss probability weighting examining ventral striatal, caudate and putamen [^11^C]MADAM binding.

We further conducted an exploratory analysis to assess the relationship between the ligand ROIs using Spearman Rank correlations (Bonferroni corrected *p* < 0.001).

## Results

### Subject characteristics

Subjects' characteristics have been previously reported (Voon et al., 2020) and are shown in Table 1. Age did not differ between groups (*p* = 0.35), though there was a group effect of body mass index (BMI) (*p* = 0.003, driven by an increased BMI in the BED population) and scores on the Beck Depression Inventory (BDI) (*p* < 0.0005, driven by higher BDI scores in both patient populations). There were also group differences across all gambling measures (driven by higher scores in the GD group) and binge eating measures (driven by higher scores in the BED group); all *p* < 0.01. Group differences in demographic and questionnaire data and ROI data, as well as within-group differences between tracer kinetics and demographic/questionnaire data were explored in previous publication (Majuri et al., 2017*a*).

**Table 1.** Demographic details of the participants

Measure	Healthy controls (*N* = 17)	BED (*N* = 7)	GD (*N* = 15)
Mean age (s.d.)	43.29 (11.10)	49.43 (5.09)	42.60 (11.81)
Males	8	0	8
Mean BMI (s.d.)	24.82 (2.10)	30.87 (6.58)	25.41 (3.64)
Mean BDI (s.d.)	2.82 (3.09)	15.43 (9.62)	14.36 (7.76)
SOGS	0.1 (0.3)	0.4 (0.5)	13.3 (2.3)
Duration of problem gambling (years)	n.a.	n.a.	11.6 (7.3)
Gambling per week (€)	3.9 (7.4)	2.9 (4.6)	152 (149)
Gambling per week (hours)	0.5 (1.2)	0.5 (1.2)	8.7 (7.2)
Gambling debt (€)	0 (0)	0 (0)	18 000 (15 600)
Binge Eating Scale	2.1 (2.1)	30.9 (4.6)	4.4 (4.4)
Yale food addiction scale	5.4 (3.4)	42.3 (6.5)	9.1 (9.5)
DEBQ emotional	20.5 (5.0)	50.0 (8.3)	21.2 (8.7)
DEBQ external	23.7 (5.3)	37.5 (6.3)	26.1 (7.3)
DEBQ restrained	24.8 (6.8)	35.3 (3.4)	20.9 (10.6)
Duration of problem eating (years)	n.a.	18.1 (14.9)	n.a.

s.d., standard deviation; BED, binge eating disorder; GD, pathological gambling; BMI, body mass index; BDI, Beck depression inventory; DEBQ, The Dutch Eating Behavior Questionnaire; SOGS, South Oaks Gambling Screen; n.a., not applicable.

### Risk-taking task behavioural results

Some participants did not complete either the risk-taking task or the PET imaging. Thus, the final analysis included (1) for the reward risk condition 15 HV, 12 GD and six BED patients and (2) for the loss risk condition 14 HV, 13 GD and six BED patients. Details can be found in Supplementary materials and methods.

No group differences were observed in risk-taking propensity between groups for rewards and losses. Mean risk-taking *w*(*p*) for HV (Reward: 0.44 (s.d. 0.22); Loss 0.49 (s.d. 0.12)], GD [Reward: 0.49 (s.d. 0.14); Loss 0.52 (s.d. 0.21)], BED [Reward: 0.31 (S 0.11); Loss 0.44 (s.d. 0.12)] (Reward *F* = 2.13, *p* = 0.13; Loss *F* = 0.55, *p* = 0.58).

### Risk-taking and PET imaging binding potential

**Fig. 3.**
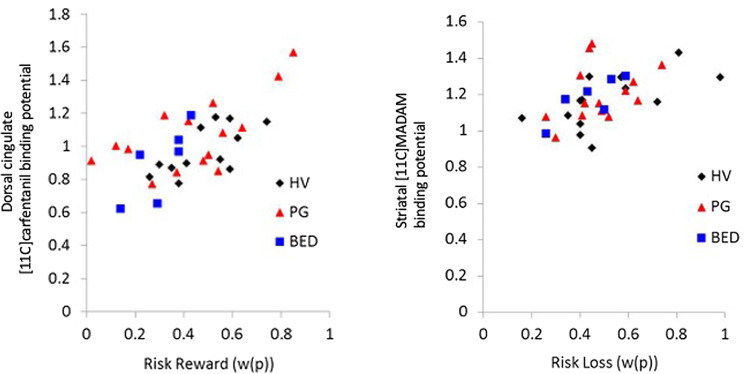
Risk-taking tendencies and neurochemical binding potential. The regression plots show the relationship between risk-taking propensities for reward (left) and loss (right) and dorsal cingulate [11C]carfentanil and striatal [11C]MADAM binding propensities respectively, in healthy volunteers (HV), pathological gamblers (PG) and binge eating disorder (BED) patients.

Risk-taking to reward was positively correlated with [^11^C]carfentanil striatum (rho = 0.548, *p* = 0.001), dorsal cingulate (rho = 0.530, *p* = 0.001) and insular binding (rho = 0.509, *p* = 0.002). Risk-taking to loss was positively correlated with [^11^C]MADAM striatal binding (rho = 0.537, *p* = 0.001) (Figure 3). No other findings were significant following stringent correction for multiple comparisons.

To measure the strength of the relationship between risk-taking to reward and observed correlations controlling for other significant correlations, we used a partial correlation analysis. Risk-taking to reward remained positively correlated with dorsal cingulate [^11^C]carfentanil binding (partial correlation coefficient =0.38, *p* = 0.027) controlling for striatal and insular binding. In contrast, neither of the other two sites were significant using partial correlation analysis: striatal [^11^C]carfentanil binding (partial correlation coefficient = 0.08, *p* = 0.65) when controlled for insular and cingulate binding and insular [^11^C]carfentanil binding (partial correlation coefficient = −0.01, *p* = 0.96) when controlled for striatal and cingulate binding.

We then analyzed the relationship between loss weightings and sub-regions of the striatum. The Spearman correlations for loss showed a significant positive correlation with [^11^C]MADAM binding for caudate (rho = 0.49, *p* = 0.004) and putamen (rho = 0.52, *p* = 0.002) but not nucleus accumbens (rho = 0.18, *p* = 0.32). We then tested ventral striatal, caudate and putamen [^11^C]MADAM binding in a single multiple regression analysis with risk-taking to loss to assess striatal specificity including caudate, putamen and ventral striatum. The best-fitting model for risk-taking to loss (*R*^2^ = 0.30, *F* = 6.55, *p* = 0.004) was positively correlated with putamen [^11^C]MADAM binding (beta = 0.32 *F* = 1.84, *p* = 0.075) and caudate [^11^C]MADAM binding (beta = 0.31 *F* = 1.79, *p* = 0.083). Mean binding potential for all three ligands in all ROIs is shown in detail in online Supplementary Table 1. Group differences in [^11^C]carfentanil BP_ND_, [^18^F]Fluorodopa *K_i_* values and [^11^C]MADAM BP_ND_ are reported in previous publications (Majuri et al., 2017*a*, *b*).

We further ran exploratory analyses to assess the relationship between ligand ROIs. As expected, we observed correlations in the relationship between ROIs for a single ligand; however, there were no significant correlations between the three different ligands for the four ROIs.

Differences in regional binding parameters between groups were not within the scope of this analysis and were addressed in previous publications (Majuri et al., 2017*a*, *b*).

## Discussion

We assessed risk-taking as a function of outcome valence across healthy controls and patient groups of behavioural addictions with PET imaging using three different ligands. We show that risk-taking for gains significantly correlated with dorsal cingulate [^11^C]carfentanil binding and risk-taking for losses correlated with dorsal striatal [^11^C]MADAM binding. Unlike these findings on risk anticipation, we have previously shown that the opioidergic and serotonergic system influence losses and rewards, respectively, in goal-directed control (Voon et al., 2020). That these neurotransmitter correlations influence opposing valences depending on the task (e.g. goal-directed control *v.* risk-taking) and also implicate differing brain regions suggests that it is unlikely that our findings solely reflect the role of valence, but rather a more complex relationship between valence and the underlying cognitive process. As our current risk anticipation task does not include feedback, it fundamentally differs from sequential learning tasks assessing goal-directed control or the use of the goal or outcome to guide choices. This current task represents underlying priors, expectations and risk biases without learning from feedback.

### Opioids in risk-taking behaviour

[^11^C]carfentanil is a selective competitive agonist to mu-opioid receptors which are distributed in multiple brain regions, including the dorsal cingulate (Vogt, Wiley, & Jensen, 1995). The dorsal cingulate is important in the representation of pain, but also in reward processing encoding the anticipation and prediction error for both rewarding and aversive outcomes. Studies have focused on the role of the mu-opioid receptors in the dorsal cingulate related to aversive processing (Zubieta et al., 2003). Surprisingly we did not observe a relationship between [^11^C]carfentanil and anticipation of risky losses in this study. The dorsal cingulate has been implicated in the anticipation of rewarding outcomes (Bush et al., 2002) and decision making in the context of uncertainty (Rogers et al., 2004). Critically, the non-selective blockade of opioid receptors with naloxone in humans decreases pleasure ratings for larger rewards associated with rostral anterior cingulate hypoactivity (Petrovic et al., 2008). Naloxone also decreases changes in sensitivity to reward value in rodents (Wassum et al., 2009). The dorsal cingulate cortex projects to the subthalamic nucleus through the hyper-direct pathway (Alexander, DeLong, & Strick, 1986), and we previously showed that deep brain stimulation targeting the subthalamic nucleus in obsessive-compulsive disorder patients reduces risk-taking specifically to reward anticipation (Voon et al., 2018). In this study, higher dorsal cingulate [^11^C]carfentanil binding can reflect either higher density of mu-opioid receptor or lower concentration of endogenous opioid peptides which compete for binding with [^11^C]carfentanil. Our findings are compatible with the interpretation that higher mu-opioid receptor density in the dorsal cingulate is associated with greater hedonic pleasure ratings to the anticipation of large reward outcomes, thus enhancing risk-taking preferences. These findings also suggest that stimulation of the dorsal cingulate mu-opioid receptor through opioid agonists may similarly influence risk-taking biases.

### Serotonergic striatal specificity and risk-taking behaviour

We show a correlation between risk-taking behaviour to avoid losses and greater dorsal striatal [^11^C]MADAM binding. Indeed, 5-HT is implicated in aversive processing and avoidance behaviour (Deakin & Graeff, 1991). Rodent studies show that lower 5-HT levels increase risk-taking (Koot et al., 2012) by decreasing preference for the safe option and increasing the subjective value of the risky option (Long et al., 2009). Similarly, rats receiving the 5-HT_1A_ receptor agonist 8-OH-DPAT fail to use combined information on the size and likelihood of future gains and losses and select more disadvantageous options in an adapted version of the human Iowa gambling task (Zeeb et al., 2009). This effect might be mediated by activation of presynaptic 5-HT_1A_ receptors and reduced global 5-HT release, or activation of postsynaptic 5-HT_1A_ receptors in 5-HT projection areas (i.e. frontal cortex) and subsequent inhibition of pyramidal cell firing.

An additional interesting finding is the striatal specificity of this correlation, with caudate and putamen [^11^C]MADAM correlating with the risk-taking behaviour in the loss domain highlighting the role of the dorsal rather than ventral striatum. We previously showed a differential involvement of prefrontal *v.* striatal [^11^C]MADAM binding in employing a habitual and goal-directed behaviour strategy accordingly (Voon et al., 2020). SERT binding may be interpreted in terms of 5-HT terminal density (SERT density), which can be either primary or adaptive in response to endogenous 5-HT level changes; these have opposing implications for 5-HT levels. High SERT density may reflect more serotonergic activity and higher SERT levels may reflect upregulation secondary to high synaptic serotonin and hence upregulation of SERT to increase synaptic reuptake. Thus, higher [^11^C]MADAM likely reflects higher serotonergic activity. Although this interpretation is not consistent with an enhanced risk-taking propensity, notably many of these studies investigate risk-taking to rewards whereas our findings are specific to losses. Thus, the enhanced serotonergic activity might decrease aversive processing, hence decreasing the aversive anticipation and possibly biasing towards greater risk-taking for losses.

### Risk-taking behaviour and DA transmission

Interestingly, we found no significant correlation between [^18^F]FDOPA binding and risk-taking behaviour, although DA is largely involved in decision-making under risk with midbrain phasic DA release relating to reward prediction errors, the difference between expected and experienced rewards (Schultz, Dayan, & Montague, 1997). [^18^F]FDOPA was originally developed to measure nigrostriatal presynaptic DA capacity (Nanni et al., 2007) although it has since been applied to measure dopaminergic transmission in both mesocortical and mesolimbic dopaminergic projections (Bragulat et al., 2007; Majuri et al., 2017*a*, *b*). Additionally, differences are observed in [^18^F]FDOPA binding in nucleus accumbens between BED patients, pathological gamblers and healthy controls (Majuri et al., 2017*a*, *b*). Also, [^18^F]FDOPA PET imaging measures presynaptic dopaminergic transmission by assessing the activity of the decarboxylating enzyme and storage capacity of DA (Nanni et al., 2007) unlike, for example, DA transporter ligands (Sekine et al., 2001). Rat studies of decision making under risk have yielded contrasting results depending on the DA receptor subtype targeted with pharmacological manipulations. For example, amphetamine, a DA releaser partially through the involvement of the DA transporter (Calipari & Ferris, 2013), both increases preference for large/risky choice (Simon et al., 2011) and decreases risky decision making (St Onge & Floresco, 2009) in separate rat studies.

Task structure might be an additional factor as Simon et al. (2011) included explicit punishment, i.e. foot shock, *v.* the non-delivery of reward applied in the study by St Onge and Floresco (2009). Also, the Iowa gambling task and the Cambridge gambling task applied in other studies involve feedback on task performance. Although the task we applied did not involve feedback, increased risk-taking only in the gain domain was shown in a previous study in healthy controls receiving L-dopa and performing a task that did not either involve learning (Rutledge et al., 2015). We also previously found a marginal relationship between goal-directed behaviour when faced with losses and putaminal [^18^F]FDOPA (Voon et al., 2020).

Thus, our findings highlight differential dorsal and ventral fronto-striatal circuitry in mediating goal-directed control and risk biases. Whereas risk-taking biases implicate dorsal striatal and dorsal cingulate regions, goal-directed control implicates nucleus accumbens and ventromedial prefrontal and medial orbitofrontal regions.

## Summary

We show distinct neurochemical substrates underlying risk-taking with the dorsal cingulate cortex mu-opioid receptor binding associated with rewards and dorsal striatal serotonin transporter binding associated with losses. We highlight distinct neurochemical and anatomical substrates as a function of valence within risk-taking and goal-directed control processes. Our findings have implications for the effects of illicit drugs and pharmaceutical agents on risk-taking tendencies and highlight the potential role of pharmacological agents or neuromodulation on modifying risk-taking biases.

## Supplementary material

For supplementary material accompanying this paper visit https://doi.org/10.1017/S0033291720005450. click here to view supplementary material
