# Supplementary material for: The neural substrates of risky rewards and losses in healthy volunteers and patient groups: a PET imaging study
Source: Psychol Med. 2021 Feb 11;52(14):3280–8. doi: 10.1017/S0033291720005450 (PMC9693671; doi:10.1017/S0033291720005450)
Supplement: Supplementary file 1 [file S0033291720005450sup001.docx]

**The neural substrates of risky rewards and losses in healthy volunteers**

**and patient groups: a PET imaging study**

**PET scanning**

One [11C]carfentanil scan and three [18F] FDOPA scans were performed on a separate day due to tracer production failure or scanner malfunction. ﻿Thus, the final sample sizes were 7 BED, 15 GD and 16 controls with [11C]carfentanil, 7 BED, 13 GD, and 16 controls with [11C]MADAM and [18F]FDOPA.

The [11C]carfentanil scans consisted of 13 frames, and the [18F]fluorodopa scans were divided into 22 frames.

**Results**

Participants not completing either condition of the risk-taking task:

One GD patient did not complete the risk reward condition, one HV did not complete the risk loss condition, one BED patient did not complete the risk reward or loss condition.

For the gain risk condition, two HV, three GD and one BED patients were not assessed in the multivariate analysis for missing data.

For the loss risk condition, three HV, two GD and one BED patients were not assessed in the multivariate analysis for missing data.

Participants not completing PET imaging:

One GD patient did not complete [18F]fluroDOPA imaging, one GD patient did not complete [11C]MADAM imaging, one GD patient and one HV did not complete [18F]fluroDOPA or [11C]MADAM imaging, and one HV did not complete [11C]carfentanil imaging.

**Table S1. Mean ligand scores across all three groups.** BP; binding potential. FDOPA; fluorodopa. Ki; ﻿influx rate constant.

| **Descriptive Statistics** | | | | | | |
| --- | --- | --- | --- | --- | --- | --- |
|  | N | Minimum | Maximum | Mean | | Std. Deviation |
|  | Statistic | Statistic | Statistic | Statistic | Std. Error | Statistic |
| Carfentanil BP insula MEAN | 38 | .5860 | 1.0946 | .856205 | .0220990 | .1362277 |
| Carfentanil BP orbitofrontal cortex MEAN | 38 | .4985 | 1.2697 | .828324 | .0278246 | .1715222 |
| Carfentanil BP striatum MEAN | 38 | .7523 | 1.5849 | 1.181403 | .0320237 | .1974073 |
| Carfentanil BP dorsal cingulate MEAN | 38 | .6254 | 1.5706 | 1.013158 | .0341885 | .2107524 |
| FPODA Ki insula MEAN | 36 | .0020 | .0034 | .002566 | .0000595 | .0003571 |
| FDOPA Ki striatum MEAN | 34 | .0039 | .0166 | .012377 | .0003303 | .0019261 |
| FDOPA Ki orbitofrontal cortex MEAN | 36 | -.0002 | .0039 | .001741 | .0001213 | .0007276 |
| FDOPA Ki dorsal cingulate MEAN | 36 | .0017 | .0045 | .003116 | .0000991 | .0005948 |
| MADAM BP insula MEAN | 36 | .4710 | .9147 | .664139 | .0152323 | .0913940 |
| MADAM BP orbitofrontal cortex MEAN | 36 | .2162 | .5972 | .398331 | .0138361 | .0830169 |
| MADAM BP striatum MEAN | 36 | .9068 | 1.4791 | 1.181558 | .0230405 | .1382429 |
| MADAM BP dorsal cingulate MEAN | 36 | .4240 | .9108 | .629611 | .0177650 | .1065902 |
| MADAM BP caudate MEAN | 36 | .5396 | 1.2836 | .909678 | .0282775 | .1696653 |
| MADAM BP putamen MEAN | 36 | 1.0561 | 1.6590 | 1.335192 | .0258649 | .1551891 |
| MADAM BP nucleus accumbens MEAN | 36 | .893 | 2.7500 | 21.509458 | .0570660 | .3423958 |
